# Construction of biocompatible bovine serum albumin nanoparticles composed of nano graphene oxide and AIEgen for dual-mode phototherapy bacteriostatic and bacterial tracking

**DOI:** 10.1186/s12951-019-0523-x

**Published:** 2019-10-10

**Authors:** Yongxin Zhang, Hao Fu, De-E Liu, Jinxia An, Hui Gao

**Affiliations:** School of Chemistry and Chemical Engineering, Tianjin Key Laboratory of Organic Solar Cells and Photochemical Conversion, Tianjin University of Technology, No. 391, West Binshui Road, Tianjin, 300384 People’s Republic of China

**Keywords:** Nano graphene oxide, Aggregation-induced emission, Dual-mode phototherapy bacteriostatic, Bacterial tracking, Biocompatibility

## Abstract

**Background:**

Efficient and highly controllable antibacterial effect, as well as good biocompatibility are required for antibacterial materials to overcome multi-drug resistance in bacteria. Herein, nano graphene oxide (NGO)-based near-infrared (NIR) photothermal antibacterial materials was schemed to complex with biocompatible bovine serum albumin (BSA) and aggregation-induced emission fluorogen (AIEgen) with daylight-stimulated ROS-producing property for dual-mode phototherapy in the treatment of antibiotic resistance bacteria.

**Results:**

Upon co-irradiation of daylight and NIR laser, NGO-BSA-AIE nanoparticles (NPs) showed superiorly antibacterial effect (more than 99%) both against amoxicillin (AMO)-resistant *Escherichia coli* (*E. coli*) and *Staphylococcus aureus* (*S. aureus*) by comparison with sing-model phototherapy. Meanwhile, the NGO-BSA-AIE NPs displayed prominent stability and excellently controllable biocompatibility. More importantly, under daylight irradiation, the AIEgen not only produced plentiful ROS for killing bacteria, but also presented fluorescence image for tracking bacteria.

**Conclusions:**

Hence, the designed system provided tempting strategy of employing light as impetus for tracking bacterial distribution and photothermal/photodynamic synergistic treatment of antibiotic resistance antibacterial. 
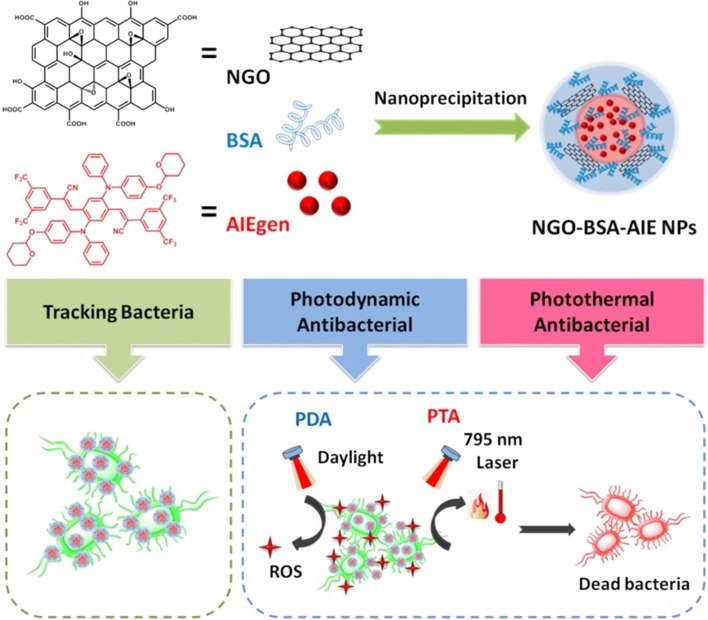

**Electronic supplementary material:**

The online version of this article (10.1186/s12951-019-0523-x) contains supplementary material, which is available to authorized users.

## Introduction

The speedy emergence of antibiotic resistant bacteria has become a global concern, and there is an urgent need for new antibacterial agent that can effectively kill antibiotic resistant bacteria [1, 2]. With the rapid development in nanotechnology, antibacterial nanomaterials have been used as alternatives to antibiotic drug [3–5]. So far, various types of antibacterial nanomaterials including organic nanomaterials and inorganic nanomaterials have been researched. For organic antibacterial nanomaterials, such as cationic polymeric nanoparticles showed excellent antibacterial effect [6–8]. Nevertheless, most of them exhibited serious cytotoxic and pro-inflammatory effect. Likewise, in pertinent to some inorganic antibacterial nanomaterial, for example gold, silver nanoparticles were reported to have high cell toxicity [9, 10]. Therefore, controllable antibacterial effect is still required, wherein the antibacterial agents keep high antibacerial effect in the pathological site, but stay well biocompatibility in normal tissue and cells.

Phototherapy is a promising modality that can be switched off/on controllably by an external light irradiation to kill bacterial [11–15]. In this technique, photothermal agents that can produce heat under the external light irradiation have received increasing attention in bacteriostatic treatment [16, 17]. Among them, graphene-based nanomaterials showed high photothermal conversion efficiency under near-infrared (NIR) light irradiation and exceptional amphiphilicity to possess a favorable affinity binding to bacterial cell membranes for the preparation of nanoscale delivery particles to encapsulate hydrophobic materials [15, 18–20]. Especially, nano graphene oxide (NGO) presented superior surface activity by comparison with micrometer-sized GO sheets [21–24]. Nevertheless, it is reported that the bacterial killing is not effective at 50–60 °C. The photothermal temperature is required up to over 70 °C to completely kill the antibiotic resistant bacteria, at which temperature the surrounding tissue and cells would be damaged [15]. Therefore, solo photothermal antibacterial method rarely accomplish precise therapy.

**Scheme 1:**
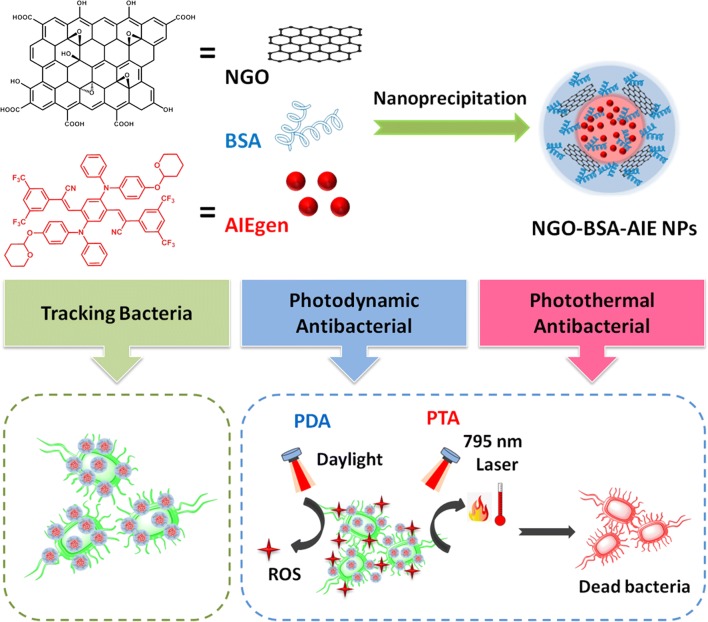
Schematic illustration of the preparation process of NGO-BSA-AIE NPs, NGO-BSA-AIE NPs for tracking bacteria and dual-mode phototherapy bacteriostatic

Except for photothermal antibacterial (PTA), another promising phototherapy for antibacterial is photodynamic antibacterial (PDA), wherein the photosensitizers produce toxic reactive oxygen species (ROS), such as singlet oxygen (^1^O_2_), superoxides and hydroxyl radicals (∙OH) to kill pathogenic bacteria on exposure to light with a suitable wavelenght [25–28]. However, most photosensitizers suffer from aggregation-caused efficacy decline. Fortunately, aggregation-induced emission fluorogen (AIEgen) is opposite to traditional organic photosensitizers and has appreciable capacity in producing ROS in their aggregated state [29, 30]. In our previous work, we developed a AIEgen, which can produce large amounts of ROS under daylight irradiation and show good fluorescent imaging in the poor solvent [31–33]. Uniting PTA with PDA is envisioned to achieve synergistic outcomes and promote enhanced bacterial ablation [16, 34]. However, the poor stability and biocompatibility of NGO/AIEgen composite in aqueous solutions are considered as obstacles for further applications in biomedical fields [35]. To address these obstacle, biocompatible bovine serum albumin (BSA) is schemed to be included the composite [36, 37]. Due to the strong hydrophobicity of AIEgen, it is proposed to entrapped it into amphiphilic NGO and BSA to form nanoparticles [35, 38]. Herein, a ternary nanoparticle (NP) formulated by NGO, BSA and AIEgen via hydrophobic interactions was developed as an antibacterial nanomaterial for dual-mode phototherapy. Dual-mode phototherapy integrating PTA with PDA is expected to produce a higher bacteriostatic efficiency through synergistic effects. More importantly, AIEgen is charecterized as not only a photosensitizer for killing bacterias, but also a fluorescence probe for tracking bacteria distribution, enabling NGO-BSA-AIE NPs to trace bacteria for better therapeutic results (Scheme 1). Overall, the proposed system provided a new platform for collaborative dual-mode phototherapy bacteriostatic and tracking bacterial distribution.

## Methods

### Preparation of NGO-BSA-AIE NPs

The BSA (2 mg) was added to NGO aqueous solution (0.5 mg mL^−1^, 1 mL) and stirred for 30 min at 25 °C followed by addition of AIEgen solution (50 μg mL^−1^, 200 μL, dissolved in THF), which was the concentration we used for antibacteria study. N_2_ was continuously passed during the reaction to remove THF. After THF was removed, the system was stirred for another 10 min to obtain NGO-BSA-AIE NPs, which was sealed and stored at 4 °C for subsequent experiments.

### Detection of ROS production

To study the ROS producing activity of the AIEgen and NGO-BSA-AIE NPs, 2′,7′-dichlorofluorescein diacetate (DCF-DA) was used to detect the production of ROS under daylight irradiation. Briefly, 0.5 mL ethanol solution of DCF-DA (1 mM) was added to 2 mL of NaOH (10 mM) aqueous solution and the mixture was stirred at 25 °C for 30 min. The hydrolysate (dichlorodihydrofluorescein, DCFH) was then neutralized with 10 mL of PBS (pH = 7.4). Then, 100 μL of obtained DCFH solution was added to 900 μL of AIEgen dispersed in THF/deionized water (1:1), as well as NGO-BSA-AIE NPs dispersed in deionized water, both of which were exposed to daylight irradiation for different time intervals at a power density of 10 mW cm^−2^ (All the light irradiation was in the same condition). The change in the fluorescence intensity of the measurement solution was performed under excitation of 488 nm while collecting emission at 500 to 600 nm.

### Photothermal effect

Firstly, 1 mL of different concentrations of NGO solution and NGO-BSA-AIE NPs were placed in a test tube. Then, the samples were irradiated with a NIR laser (795 nm) at a power density of 2.5 W cm^−2^ for 10 min.

### Antibacterial activity

AMO^r^ *E. coli* and AMO^r^ *S. aureus* were inoculated into LB liquid medium, and after incubating overnight at 37 °C in a shaking incubator (170 rpm), the bacteria were centrifuged (8000 rpm, 5 min) and suspended in PBS buffer solution (pH = 7.4). Subsequently, the bacterial suspension was serially dilutedwith PBS to a concentration of 10^6^ CFU mL^−1^. In detail, diluted bacterial suspensions (AMO^r^ *E. coli* and AMO^r^ *S. aureus*) without any treatment were used as a control. Furthermore, the bacterial suspension treated with AMO (100 μg mL^−1^ for AMO^r^ *E. coli* and AMO^r^ *S. aureus*) were to demonstrate the antibiotic resistance of the bacteria. Both blank bacterial suspensions and bacterial suspensions treated with NGO-BSA-AIE NPs were exposed to daylight for 1 h followed by NIR irradiation for 5 min (795 nm, 2.5 W cm^−2^). While the bacterial suspensions treated with NGO-BSA-AIE NPs were stored in a dark environment, or bacterial irradiated with daylight for 1 h, or irradiated with 795 nm NIR laser for 5 min. All of the suspensions were cultured in the incubator for 4 h, followed by being diluted with an appropriate dilution factor. The diluted treated bacterial suspensions (100 μL) were transferred to solid LB agar plates, then incubated at 37 °C for 16 h. After the cultivation, the number of colony forming units (CFU) was counted, and the bacterial survival rates and antibacterial efficiency were measured.

The CFU ratio was calculated using the following equation: CFU ratio = C/C_0_ × 100%. C and C_0_ were the CFU of the experimental group treated with NGO-BSA-AIE NPs or the control group without any treatments, respectively. Results were expressed as the mean and standard deviation of three parallel groups.

And the antibacterial efficiency was calculated using the following equation: antibacterial efficiency = (1 − C/C_0_) × 100%.

### Bacterial imaging

AMO^r^ *E. coli* and AMO^r^ *S. aureus* were incubated at 37 °C overnight to a concentration of 10^9^ CFU mL^−1^ and washed with PBS three times, followed by being transferred to a 35 mm glass-bottom after proper dilution. NGO-BSA-AIE NPs (100 μL) were then added to the plate. After 6 h of incubation, the bacterial suspensions were washed three times with PBS buffer, and finally the bacteria were retained in 1 mL of PBS for bioimaging.

### Cytotoxicity study

MTT assay was performed in this experiment to assess the cell viability. L929 cells were seeded in 96-well U-bottom plates at a density of 5000 cells per well and incubated at 37 °C for 24 h in culture medium. After overnight incubation, cells were treated with different concentrations of NGO-BSA-AIE NPs. Then replaced the fresh cell medium and further cultured for 24 h. After that, MTT solution (5 mg mL^−1^) was added to 96-well plates at 10 µL per well and incubated for 4 h. After removing MTT solution, 100 μL of filtered DMSO was added into each well to dissolve all the formed crystals. The cell viability was accessed by means of MTT absorbance at 570 nm recorded using a microplate reader (Epoch, BioTek, Gene company Limited). The cell viability in each well was calculated from the obtained values as a percentage of control wells. The results were presented as a mean and standard deviation obtained from eight samples.

### Statistics analysis

Significant differences in bacterial viability between any two groups were assessed using Student’s *t* test.

## Results and discussion

### Synthesis and characterization of NGO

**Fig. 1:**
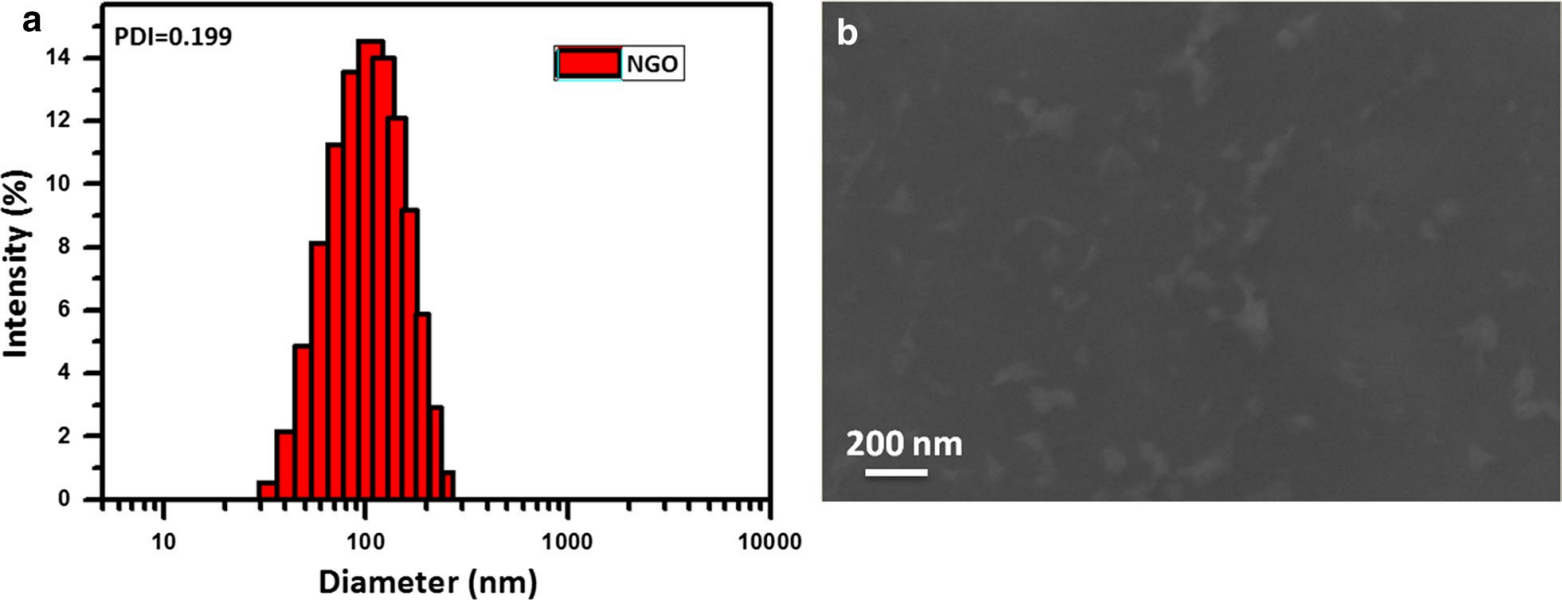
**a** Size distribution and **b** SEM image of synthetic NGO

Firstly, GO was synthesized using natural graphite by an improved Hummer’s method [22, 39]. The resulting GO was characterized by X-ray diffraction (XRD), Fourier transform infrared (FT-IR) measurements and scanning electron microscopy (SEM) images (Additional file 1: Figure S1). These results indicated the successful preparation of GO. Afterwards, GO was sonicated in water and filtered to obtain NGO. The obtained NGO displayed a particle size of 93 nm with polydispersity index (PDI = 0.199) via dynamic light scattering (DLS) measurment (Fig. 1a). SEM was further utilized to check the morphology of NGO. As shown in Fig. 1b, the NGO presented as nanoparticles with average diameter of 85 ± 15.5 nm, which is consist with the result of DLS measurments.

### Synthesis and characterization of AIEgen

**Fig. 2:**
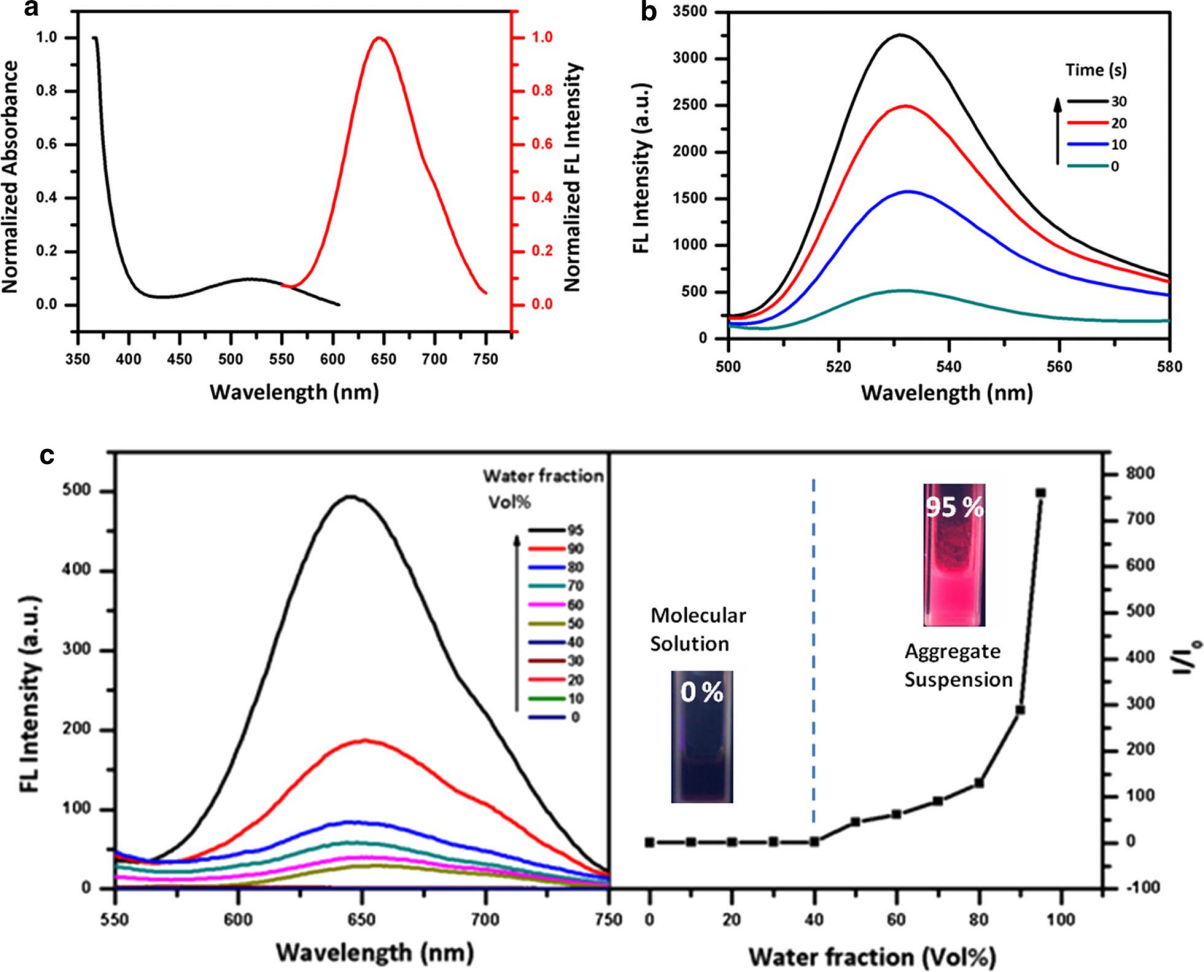
**a** Absorption and emission spectra of the synthesized AIEgen. **b** FL spectra of the AIEgen and DCF mixture in THF/water to detect its ROS production under daylight exposures (10 mW cm^−2^) for various time period. **c** Aggregation-induced emission characteristics of AIEgen: FL spectra of AIEgen in different ratios of THF/water mixture (λex = 493 nm) and calculated I/I_0_ ratios as a function of water fraction

The AIEgen used in the present study was synthesized according to the previously reported method [31–33]. Successful synthesis of AIEgen was verified by ^1^H-NMR measurement. Afterwards, we examined the UV–vis/fluorescence spectroscopic and ROS-producing activity of AIEgen. As shown in Fig. 2a, the as-synthesized AIEgen had an excitation wavelength of approximately 500 nm, while the emission wavelength appeared to be in the range of 600–750 nm with an emission maximum at 640 nm, which was in the red/NIR region and suggested its potential application with considerable tissue penetration. Figure 2c showed the unique AIE characteristics of the proposed AIEgen. The fluorescence intensity (FL) increased rapidly with the addition of water. Then, the ROS-producing activity of the synthesized AIEgen under daylight (10 mW cm^−2^) for various time period was detected using dichlorofluorescein (DCF) as an indicator (Fig. 2b). The experimental results confirmed that AIEgen produced large amounts of ROS with the extension of illumination time, demonstrating that AIEgen can be used as an effective ROS self-sufficient component.

### Fabrication and characterization of NGO-BSA-AIE NPs

**Fig. 3:**
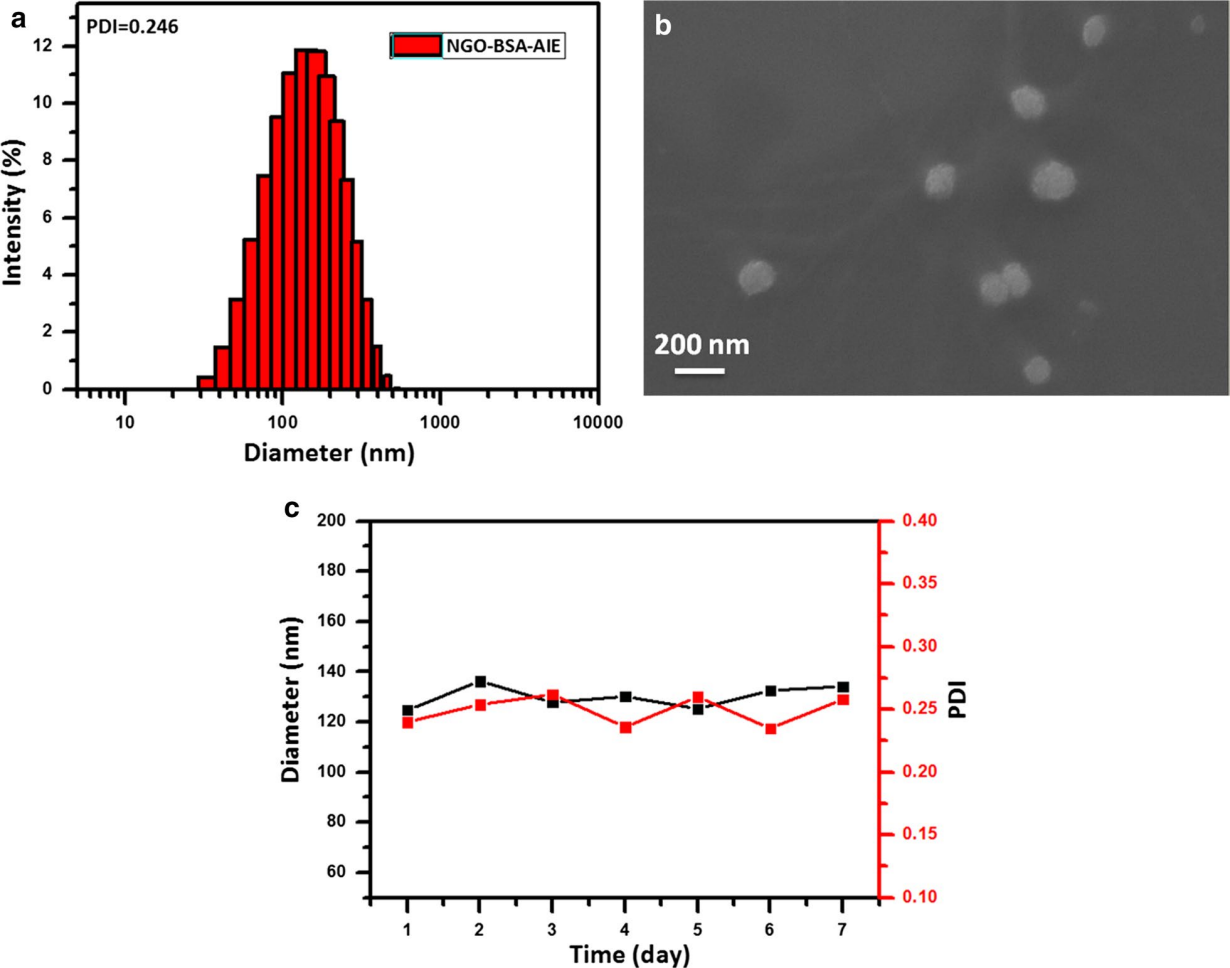
Characterization of NGO-BSA-AIE NPs by **a** DLS, **b** SEM imaging (Scale bars: 200 nm) and **c** Stability evaluation of NGO-BSA-AIE NPs within 7 days in RPMI 1640 media by DLS size monitoring

NGO-BSA-AIE NPs were prepared by nanoprecipitation method [29]. Briefly, AIEgen solution (50 μg mL^−1^, 200 μL, dissolved in THF) was added to the mixed solution of BSA (2 mg mL^−1^) and NGO (0.5 mg mL^−1^), then N_2_ was continuously passed during the reaction to remove THF and NGO-BSA-AIE NPs was obtained. The resulting NGO-BSA-AIE NPs showed a hydrodynamic diameter of approximately 125 nm with a PDI of 0.246 as determined by DLS measurements (Fig. 3a). SEM was further performed to observe the morphology of NGO-BSA-AIE NPs. It presented as spherical morphology with a diameter of 105 ± 12.5 nm (Fig. 3b). Notably, individual NGOs were very unstable in salt solutions (Additional file 1: Figure S2a), which was in agreement with previous reports [35]. Then, the stability of NGO-BSA-AIE NPs in cell medium, saline aqueous solution (PBS) and water were checked via DLS measurements. As confirmed in Fig. 3c and Additional file 1: Figure S2, the obtained NGO-BSA-AIE NPs exhibited significant stability with consistent hydrodynamic diameter and PDI over a 7-day continuous measurements, showing the potential application in biomedical therapeutics.

### The photodynamic properties

**Fig. 4:**
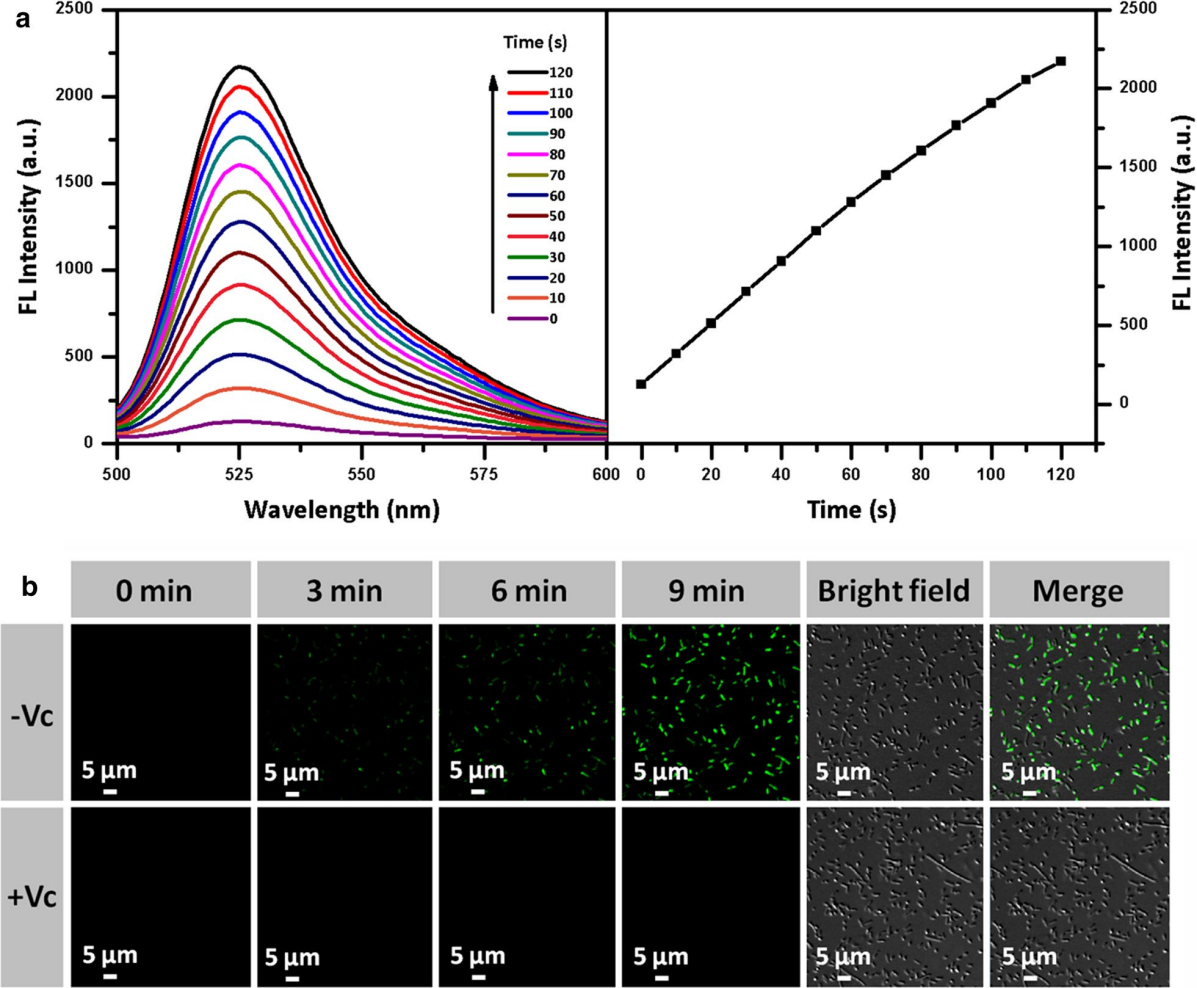
**a** ROS production under daylight exposures (10 mW cm^−2^). **b** CLSM imaging of AMO^r^ *E. coli* after incubation with NGO-BSA-AIE NPs and DCF-DA under daylight irradiation for different time in the presence and absence of vitamin C. (λex = 488 nm, λem = 525 nm) Scale bars: 5 μm

The ROS-producing activity of NGO-BSA-AIE NPs under daylight exposures (10 mW cm^−2^) was detected using DCF as an indicator [33]. The fluorescence intensity increased with the prolonged time of daylight irradiation, which was beneficial to control the antibacterial photodynamic therapy (Fig. 4a). We further explored the production of ROS from NGO-BSA-AIE NPs in AMO^r^ *E. coli* (Fig. 4b) and AMO^r^ *S. aureus* (Additional file 1: Figure S3) according to confocal laser scanning microscopy (CLSM). Remarkable ROS was detected under light irradiation with the aid of DCF-DA indicator, and the green fluorescence intensity became stronger with extended daylight exposure time, indicating that the amount of ROS production in the bacteria increased with the irradiation time. The control group was treated with vitamin C because it had a strong antioxidant effect, thereby inhibiting the production of ROS in bacteria by NGO-BSA-AIE NPs. Therefore, NGO-BSA-AIE NPs have intriguing potential for effective antibacterial photodynamic therapy.

### The photothermal properties

**Fig. 5:**
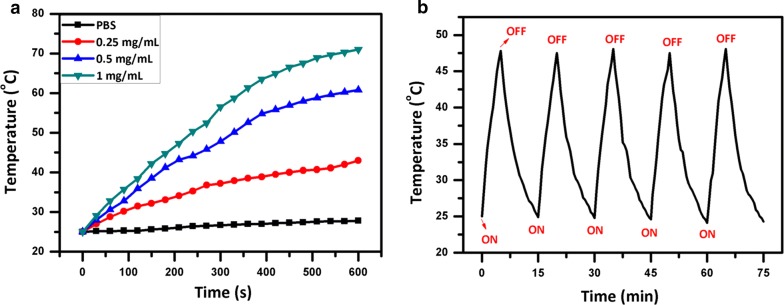
**a** Photothermal curves of different concentrations of NGO-BSA-AIE NPs under 795 nm NIR laser irradiation. NGO: 0.25, 0.5, 1.0 mg mL^−1^; BSA: 1.0, 2.0, 4.0 mg mL^−1^; AIE: 0.5, 10, 20 μg mL^−1^. **b** The temperature rising and cooling curve of the NGO-BSA-AIE NPs with 5 times laser switch-on and switch-off treatment

GO has strong optical absorption in the NIR region (700–1100 nm) and can be used as a photothermal agent [26, 40]. Besides efficient photodynamic activity, NGO-BSA-AIE NPs is anticipated to possess exceptional photothermal properties. Figure 5a showed the temperature change of different concentrations of NGO-BSA-AIE NPs under 795 nm NIR laser irradiation. The NGO-BSA-AIE NPs exhibited an obvious temperature elevation of 14 °C within 10 min at the NGO concentration of 0.25 mg mL^−1^. Higher concentration of NGO led to enhanced temperature increase. It is worth noting that both NGO and NGO-BSA-AIE NPs of the same concentration exhibited the same photothermal effect, indicating that inclusion of AIEgen and BSA did not influence the photothermal performance of NGO (Additional file 1: Figure S4). Additionally, to test the photothermal stability of NGO-BSA-AIE NPs, repeated laser on and off were performed. As shown in Fig. 5b, the temperature of the NGO-BSA-AIE NPs solution exhibited a significant switching effect with or without NIR laser irradiation. Even after being treated for 5 cycles, NGO-BSA-AIE NPs could still rise to about 50 °C upon exposure to NIR laser for 5 min, demonstrating excellent photothermal stability.

### Antibacterial activity

**Fig. 6:**
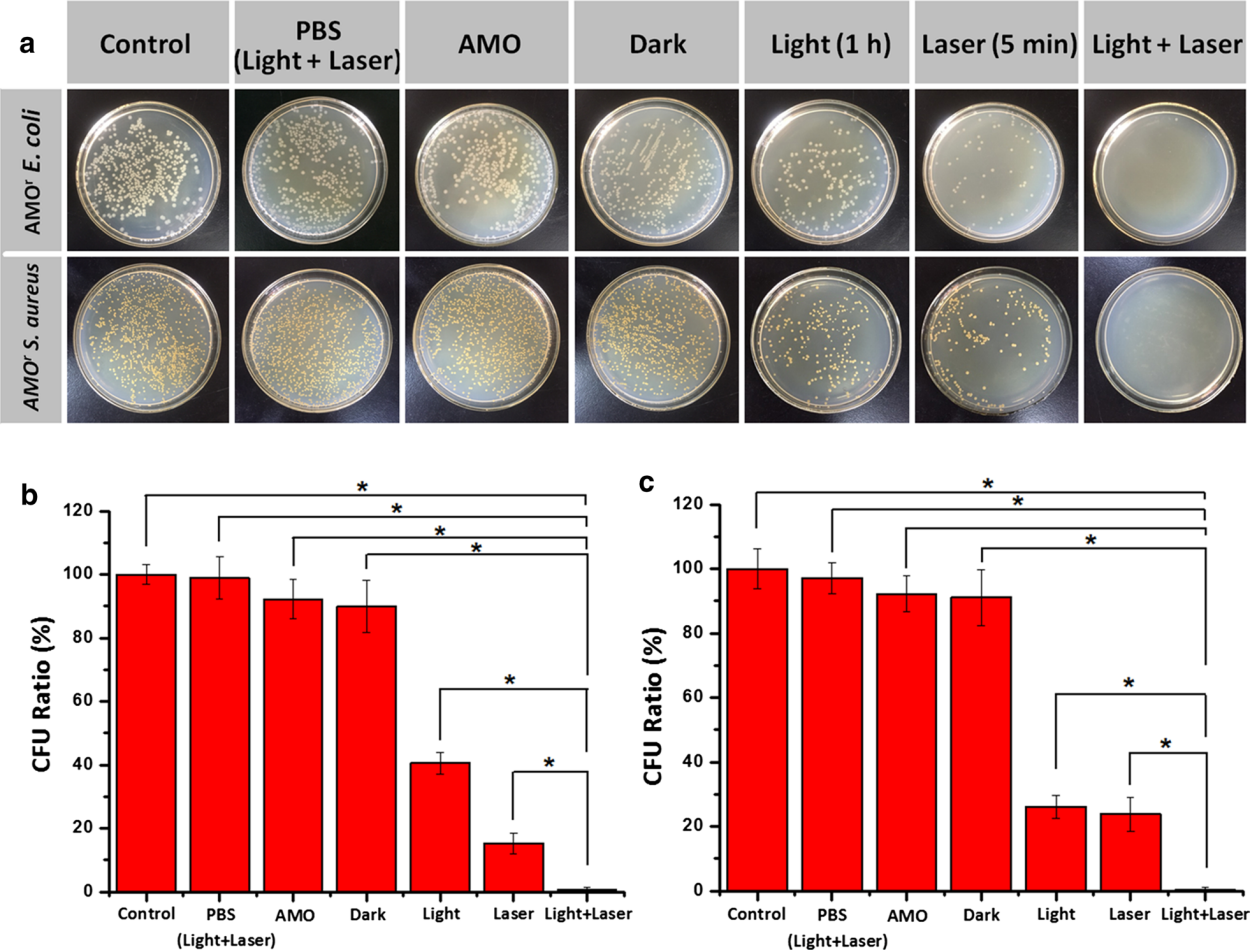
**a** CFU for AMO^r^ *E. coli* and AMO^r^ *S. aureus* of control group without any treatment, bacterial in PBS that exposed to daylight for 1 h (10 mW cm^−2^) followed by NIR irradiation for 5 min (795 nm, 2.5 W cm^−2^), NGO-BSA-AIE NPs that presented in a dark environment or irradiated with light/laser. Quatitative results of CFU of **b** AMO^r^ *E. coli* and **c** *AMO*^*r*^ *S. aureus*. Significant differences between every two groups (p < 0.05) are indicated by an asterisk (*)

The antibacterial property of NGO-BSA-AIE NPs combining PTA with PDA was evaluated. Herein, the culture was passaged 30 times in a continuous treatment in which the concentration of AMO was gradually increased to obtain AMO^r^ *E. coli* and AMO^r^ *S. aureus* [5]. To confirm the antibiotic resistance, the bacterial suspensions treated with AMO (100 μg mL^−1^) showed a survival rate of 92.2% for AMO^r^ *E. coli* and 92.1% for AMO^r^ *S. aureus*. Figure 6 showed the antibacterial effect of NGO-BSA-AIE NPs against AMO^r^ *E. coli* and AMO^r^ *S. aureus* with or without daylight and NIR laser irradiation. Diluted bacterial (PBS, pH = 7.4) suspensions without any treatment were used as control. According to the spread-plate results, the CFU was counted in the bacterial plate to evaluate the survival rates and antibacterial efficiency (Fig. 6a–c). Obviously, NGO-BSA-AIE NPs showed neglectable antibacterial efficiency in the absence of light irradiation. The bacterial survival rates of AMO^r^ *E. coli* and AMO^r^ *S. aureus* were 89.9% and 91.1%, respectively. Meanwhile, blank bacterial suspensions treated with daylight and NIR laser irradiation confirmed that the irradiation treatment did not affect the bacteriostatic efficiency, which showed a survival rate of 98.8% and 97.1% for AMO^r^ *E. coli* and AMO^r^ *S. aureus*. Whereas, the antibacterial efficiency of bacterial suspensions treated with NGO-BSA-AIE NPs after exposure to daylight and NIR irradiation against AMO^r^ *E. coli* and AMO^r^ *S. aureus* was both over 99%. By comparison, bacterial suspensions treated with NGO-BSA-AIE NPs under exposure to daylight or NIR irradiation displayed faint inhibitory effects on AMO^r^ *E. coli* (40.5%, 15.3% of CFU ratio) and AMO^r^ *S. aureus* (26.1%, 23.8% of CFU ratio). Complete antibacterial efficiency was rarely fulfilled under solo laser irradiation. Therefore, compared with other nanoparticles using solo PTA or PDA, [41, 42] the dual-mode phototherapy antibacterial treatment of NGO-BSA-AIE NPs achieved high antibacterial effect while avoiding the high temperature associated with continuous photothermal therapy that could damage the surrounding normal tissues and cells, as well as the side effects of continuous photodynamic therapy. In addition, the efficient inhibition of NGO-BSA-AIE NPs against antibiotic-resistant bacteria has a great potential to overcome multi-drug resistance in bacteria by comparison with other reported antibacterial particles [43, 44].

**Fig. 7:**
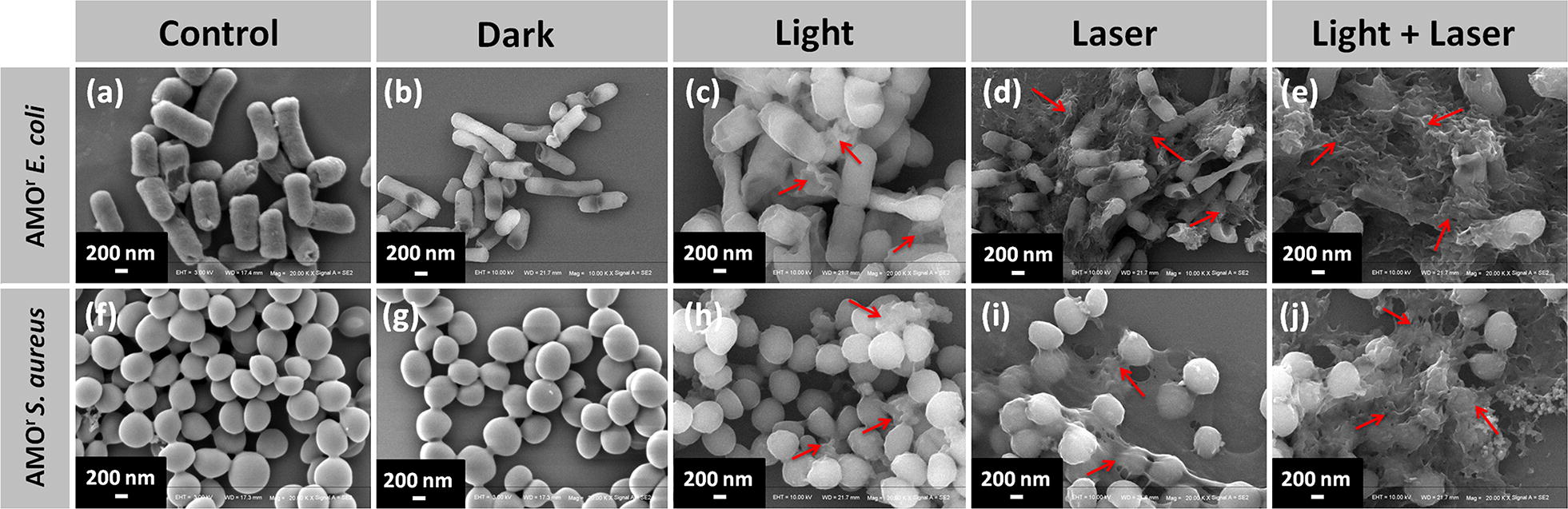
SEM images of AMO^r^ *E. coli* and AMO^r^ *S. aureus* treated with PBS (**a**, **f**) and NGO-BSA-AIE NPs that presented in a dark environment (**b**, **g**), irradiated with daylight (**c**, **h**), irradiated with 795 nm NIR laser (**d**, **i**) and irradiated with daylight followed by 795 nm NIR laser (**e**, **j**). Scale bars: 200 nm

Afterwards, we further evidence the antimicrobial behavior of NGO-BSA-AIE NPs by SEM and CLSM. As shown in Fig. 7, the bacteria treated with NGO-BSA-AIE NPs were observed to exhibit the same smooth cell membrane margins and intact bacterial morphology as control group (Fig. 7a, f), while the morphology of the bacteria changed slightly upon daylight or NIR laser irradiation, with part of bacterial deformed and collapsed (indicated by the red arrow). More interestingly, AMO^r^ *E. coli* and AMO^r^ *S. aureus* treated with the NGO-BSA-AIE NPs were found to show significant deformation with their cell membranes completely collapse after exposure to both light irradiation, verifying high antibacterial effect under dual-model phototherapy.

Likewise, similar result was observed via CLSM (Additional file 1: Figure S5a, b). The survival of bacteria were evaluated by acridine orange (AO) and ethidium bromide (EB) stains. AO was empolyed to stain living bacteria and produce green fluorescence, while EB was utilized to stain dead bacteria and produce red fluorescence. As expected, the bacteria (AMO^r^ *E. coli* and AMO^r^ *S. aureus*) in the control group showed green fluorescence entirely, while the bacteria incubated with the NGO-BSA-AIE NPs irradiated with daylight and NIR laser almost presented red fluorescence, confirming significant antibacterial efficiency. These results certified that NGO-BSA-AIE NPs can achieve more efficacious bacteriostatic effect under collaborative dual-mode phototherapy.

### Bacterial imaging

**Fig. 8:**
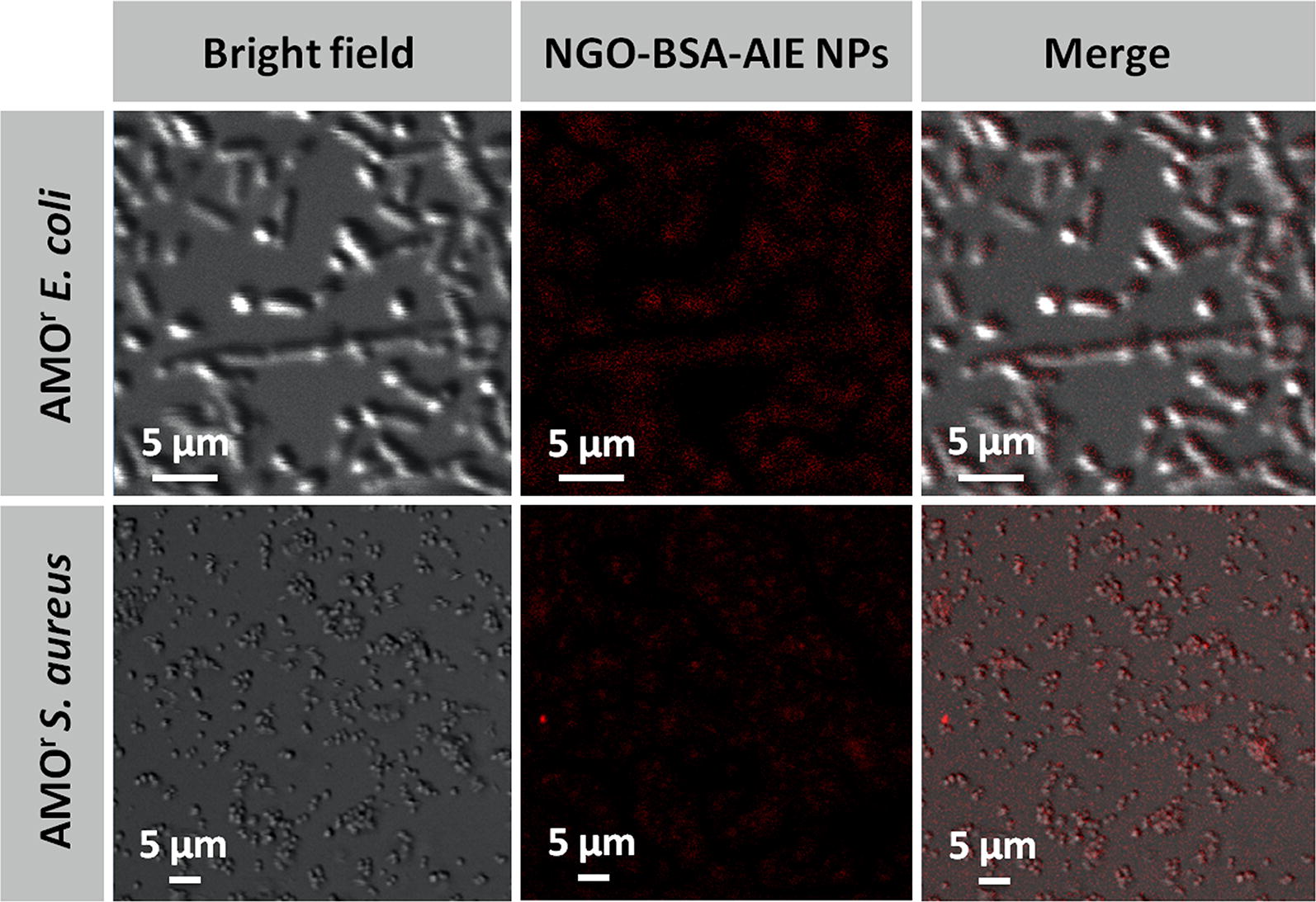
Intracellular distribution observed by CLSM of NGO-BSA-AIE NPs in AMO^r^ *E. coli* and AMO^r^ *S. aureus*. Scale bars: 5 μm

AIEgen was supposed to be applied as bacterial tracer because of its red/NIR emission characteristics. The feasibility of the proposed NGO-BSA-AIE NPs as potential bacterial tracer was verfied by CLSM measurements on bacteria (AMO^r^ *E. coli* and AMO^r^ *S. aureus*). As shown in Fig. 8, NGO-BSA-AIE NPs appeared to be internalized into bacteria, and red fluorescence of NGO-BSA-AIE NPs was present in the bacterial cytoplasm, confirming that the proposed NGO-BSA-AIE NPs have great potential as fluorescent reporters for bacterial imaging, which is beneficial for observing the of bacterial distribution. In order to further understand the interaction between NGO-BSA-AIE NPs and cells, we conducted CLSM measurement for the eukaryotic cells (L929 cells) incubated with the proposed NGO-BSA-AIE NPs. As shown in Additional file 1: Figure S6, consistent with the experimental results of bacterial imaging, NGO-BSA-AIE NPs were clearly observed to be internalized into L929 cells.

### Biocompatibility evaluation

**Fig. 9:**
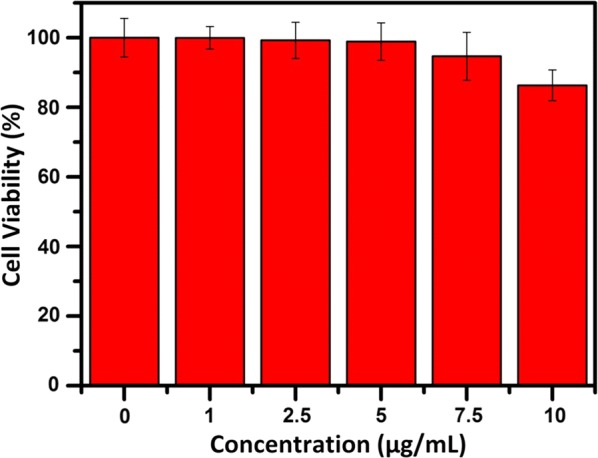
Cell viability assay of L929 cells treated with different concentrations of AIEgen of NGO-BSA-AIE NPs

Obviously, the biocompatibility of antibacterial agents is the major concern for their practical applications, especially the cytotoxicity to normal cells [45]. Herein, cytotoxicity of different concentrations of NGO BSA-AIE NPs on fibroblast L929 was tested by commercially available MTT assays. As shown in Fig. 9, even if the concentration of AIEgen was increased to 10 μg/mL (the concentration we used for antibacteria study), the cell viability was still above 85%. Therefore, the proposed NGO-BSA-AIE NPs exhibited low cytotoxicity, thereby indicating its intriguing potential in biomedical practical applications. Moreover, compared to other high cytotoxic and proinflammatory antibacterial nanoparticles such as cationic polymers [7, 8] and Au, Ag nanoparticles, [9, 10] etc., the experiment results demonstrated that NGO-BSA-AIE NPs have the advantage of exhibiting a high antibacterial effect at the pathological site but still maintaining good biocompatibility in normal cells.

## Conclusions

In summary, we have fabricated a novel antibacterial nanoparticle composed of NGO, BSA and AIEgen through a simple nanoprecipitation method. NGO-BSA-AIE NPs can produce moderate amount of heat under NIR laser irradiation and produce abundant ROS under daylight to destroy bacteria. NGO-BSA-AIE NPs showed high and controlled antibacterial efficiency, as well as good biocompatibility. The antibacterial efficiency of NGO-BSA-AIE NPs after exposure to daylight and NIR irradiation was both over 99%, but low cytotoxicity was found in the dark condition. In addition, due to the NIR emission characteristics of AIEgen, the bacteria treated with NGO-BSA-AIE NPs showed red fluorescence under CLSM, demonstrating that the NPs can be applied in the field of bacterial tracer. Hence, the proposed NGO-BSA-AIE NPs provided an important platform for the synergistic bacteriostatic effect through photodynamic and photothermal therapy, as well as NIR image tracer in biomedical applications.

## Additional file

**Additional file 1.** Additional experimental section and additional figures.

## Data Availability

All data and material are included in the article and its additional files.

## Abbreviations

NGO: nano graphene oxide
NIR: near-infrared
BSA: bovine serum albumin
AIEgen: aggregation-induced emission fluorogen
AMO^r^: amoxicillin-resistant
NPs: nanoparticles
ROS: reactive oxygen species
PTA: photothermal antibacterial
PDA: photodynamic antibacterial
DCF: dichlorofluorescein
DCF-DA: 2′,7′-dichlorofluorescein diacetate
DCFH: dichlorodihydrofluorescein
CFU: colony forming units
XRD: X-ray diffraction
FT-IR: Fourier transform infrared
SEM: scanning electron microscopy
DLS: dynamic light scattering
CLSM: confocal laser scanning microscopy
AO: acridine orange
EB: ethidium bromide
MTT: 3-(4,5-dimethyl thiazol-2-yl)-2,5-diphenyltetrazolium bromide

## References

[1] Sun M, Qu A, Hao C, Wu X, Xu L, Xu C, Kuang H (2018). Chiral upconversion heterodimers for quantitative analysis and bioimaging of antibiotic-resistant bacteria in vivo. Adv Mater.

[2] Galvan DD, Yu Q (2018). Surface-enhanced raman scattering for rapid detection and characterization of antibiotic-resistant bacteria. Adv Healthc Mater.

[3] Ren S, Boo C, Guo N, Wang S, Elimelech M, Wang Y (2018). Photocatalytic reactive ultrafiltration membrane for removal of antibiotic resistant bacteria and antibiotic resistance genes from wastewater effluent. Environ Sci Technol.

[4] Yang Y, He P, Wang Y, Bai H, Wang S, Xu JF, Zhang X (2017). Supramolecular radical anions triggered by bacteria in situ for selective photothermal therapy. Angew Chem Int Ed Engl.

[5] Chen S, Chen Q, Li Q, An J, Sun P, Ma J, Gao H (2018). Biodegradable synthetic antimicrobial with aggregation-induced emissive luminogens for temporal antibacterial activity and facile bacteria detection. Chem Mater.

[6] Alonso A, Muñoz-Berbel X, Vigués N, Rodríguez-Rodríguez R, Macanás J, Muñoz M, Mas J, Muraviev DN (2013). Superparamagnetic Ag@Co-nanocomposites on granulated cation exchange polymeric matrices with enhanced antibacterial activity for the environmentally safe purification of water. Adv Funct Mater.

[7] Ding X, Duan S, Ding X, Liu R, Xu F-J (2018). Versatile antibacterial materials: an emerging arsenal for combatting bacterial pathogens. Adv Funct Mater.

[8] Lin J, Chen X, Chen C, Hu J, Zhou C, Cai X, Wang W, Zheng C, Zhang P, Cheng J (2018). Durably antibacterial and bacterially antiadhesive cotton fabrics coated by cationic fluorinated polymers. ACS Appl Mater Interfaces.

[9] Bar-Ilan O, Albrecht RM, Fako VE, Furgeson DY (2009). Toxicity assessments of multisized gold and silver nanoparticles in zebrafish embryos. Small.

[10] Xiong Y, Brunson M, Huh J, Huang A, Coster A, Wendt K, Fay J, Qin D (2013). The role of surface chemistry on the toxicity of ag nanoparticles. Small.

[11] Tan X, Wang J, Pang X, Liu L, Sun Q, You Q, Tan F, Li N (2016). Indocyanine green-loaded silver nanoparticle@polyaniline core/shell theranostic nanocomposites for photoacoustic/near-infrared fluorescence imaging-guided and single-light-triggered photothermal and photodynamic therapy. ACS Appl Mater Interfaces.

[12] Wang YY, Wang WL, Shen XC, Zhou B, Chen T, Guo ZX, Wen CC, Jiang BP, Liang H (2018). Combination-responsive MoO_3-x_ hybridized hyaluronic acid hollow nanospheres for cancer phototheranostics. ACS Appl Mater Interfaces.

[13] Luo G-F, Chen W-H, Lei Q, Qiu W-X, Liu Y-X, Cheng Y-J, Zhang X-Z (2016). A Triple-collaborative strategy for high-performance tumor therapy by multifunctional mesoporous silica-coated gold nanorods. Adv Funct Mater.

[14] Yu S, Li G, Liu R, Ma D, Xue W (2018). Dendritic Fe3O4@poly(dopamine)@PAMAM nanocomposite as controllable NO-releasing material: a synergistic photothermal and NO antibacterial study. Adv Funct Mater.

[15] Li Yuan, Tan Lei, Cui Zhenduo, Yang Xianjin, Zheng Yufeng, Yeung Kelvin Wai Kwok, Chu Paul K, Shuilin Wu (2018). Rapid sterilization and accelerated wound healing using Zn^2+^ and graphene oxide modified g-C3N4 under dual light irradiation. Adv Funct Mater.

[16] Li L, Liu Y, Hao P, Wang Z, Fu L, Ma Z, Zhou J (2015). PEDOT nanocomposites mediated dual-modal photodynamic and photothermal targeted sterilization in both NIR I and II window. Biomaterials.

[17] Gao DY, Ji X, Wang JL, Wang YT, Li DL, Liu YB, Chang KW, Qu JL, Zheng J, Yuan Z (2018). Engineering a protein-based nanoplatform as an antibacterial agent for light activated dual-modal photothermal and photodynamic therapy of infection in both the NIR I and II windows. J Mater Chem B.

[18] Li M, Yang X, Ren J, Qu K, Qu X (2012). Using graphene oxide high near-infrared absorbance for photothermal treatment of Alzheimer’s disease. Adv Mater.

[19] Pan J, Yang Y, Fang W, Liu W, Le K, Xu D, Li X (2018). Fluorescent phthalocyanine–graphene conjugate with enhanced NIR absorbance for imaging and multi-modality therapy. ACS Appl Nano Mater.

[20] Ran X, Du Y, Wang Z, Wang H, Pu F, Ren J, Qu X (2017). Hyaluronic acid-templated Ag nanoparticles/graphene oxide composites for synergistic therapy of bacteria infection. ACS Appl Mater Interfaces.

[21] Diez-Pascual AM, Diez-Vicente AL (2016). Poly(propylene fumarate)/polyethylene glycol-modified graphene oxide nanocomposites for tissue engineering. ACS Appl Mater Interfaces.

[22] Dellieu L, Lawarée E, Reckinger N, Didembourg C, Letesson JJ, Sarrazin M, Deparis O, Matroule JY, Colomer JF (2015). Do CVD grown graphene films have antibacterial activity on metallic substrates?. Carbon.

[23] Fan Z, Liu B, Wang J, Zhang S, Lin Q, Gong P, Ma L, Yang S (2014). A novel wound dressing based on Ag/graphene polymer hydrogel: effectively kill bacteria and accelerate wound healing. Adv Funct Mater.

[24] Hui L, Huang J, Chen G, Zhu Y, Yang L (2016). Antibacterial property of graphene quantum dots (both source material and bacterial shape matter). ACS Appl Mater Interfaces.

[25] Zhang K, Meng X, Cao Y, Yang Z, Dong H, Zhang Y, Lu H, Shi Z, Zhang X (2018). Metal-organic framework nanoshuttle for synergistic photodynamic and low-temperature photothermal therapy. Adv Funct Mater.

[26] Lu Y, Li L, Lin Z, Wang L, Lin L, Li M, Zhang Y, Yin Q, Li Q, Xia H (2018). A new treatment modality for rheumatoid arthritis: combined photothermal and photodynamic therapy using Cu_7.2_S_4_ nanoparticles. Adv Healthc Mater.

[27] Li W, Peng J, Tan L, Wu J, Shi K, Qu Y, Wei X, Qian Z (2016). Mild photothermal therapy/photodynamic therapy/chemotherapy of breast cancer by Lyp-1 modified docetaxel/IR820 co-loaded micelles. Biomaterials.

[28] Feng Z, Liu X, Tan L, Cui Z, Yang X, Li Z, Zheng Y, Yeung KWK, Wu S (2018). Electrophoretic deposited stable chitosan@MoS_2_ coating with rapid in situ bacteria-killing ability under dual-light irradiation. Small.

[29] Zhu Z, Qian J, Zhao X, Qin W, Hu R, Zhang H, Li D, Xu Z, Tang BZ, He S (2016). Stable and size-tunable aggregation-induced emission nanoparticles encapsulated with nanographene oxide and applications in three-photon fluorescence bioimaging. ACS Nano.

[30] Wang H, Ma K, Xu B, Tian W (2016). Tunable supramolecular interactions of aggregation-induced emission probe and graphene oxide with biomolecules: an approach toward ultrasensitive label-free and “Turn-On” DNA sensing. Small.

[31] Lu H, Zheng Y, Zhao X, Wang L, Ma S, Han X, Xu B, Tian W, Gao H (2016). Highly efficient far red/near-infrared solid fluorophores: aggregation-induced emission, intramolecular charge transfer, twisted molecular conformation, and bioimaging applications. Angew Chem Int Ed Engl.

[32] Guan Y, Lu H, Li W, Zheng Y, Jiang Z, Zou J, Gao H (2017). Near-infrared triggered upconversion polymeric nanoparticles based on aggregation-induced emission and mitochondria targeting for photodynamic cancer therapy. ACS Appl Mater Interfaces.

[33] Huang Y, Chen Q, Lu H, An J, Zhu H, Yan X, Li W, Gao H (2018). Near-infrared AIEgen-functionalized and diselenide-linked oligo-ethylenimine with self-sufficing ROS to exert spatiotemporal responsibility for promoted gene delivery. J Mater Chem B.

[34] Hu X, Tian H, Jiang W, Song A, Li Z, Luan Y (2018). Rational design of IR820-and Ce6-based versatile micelle for single NIR laser-induced imaging and dual-modal phototherapy. Small.

[35] Sun X, Zebibula A, Dong X, Zhang G, Zhang D, Qian J, He S (2018). Aggregation-induced emission nanoparticles encapsulated with PEGylated nano graphene oxide and their applications in two-photon fluorescence bioimaging and photodynamic therapy in vitro and in vivo. ACS Appl Mater Interfaces.

[36] Li Z, Yang T, Lin C, Li Q, Liu S, Xu F, Wang H, Cui X (2015). Sonochemical synthesis of hydrophilic drug loaded multifunctional bovine serum albumin nanocapsules. ACS Appl Mater Interfaces.

[37] Zeng Q, Zhang R, Zhang T, Xing D (2019). H_2_O_2_-responsive biodegradable nanomedicine for cancer-selective dual-modal imaging guided precise photodynamic therapy. Biomaterials.

[38] Qin W, Ding D, Liu J, Yuan WZ, Hu Y, Liu B, Tang BZ (2012). Biocompatible nanoparticles with aggregation-induced emission characteristics as far-red/near-infrared fluorescent bioprobes for in vitro and in vivo imaging applications. Adv Funct Mater.

[39] Dikin DA, Stankovich S, Zimney EJ, Piner RD, Dommett GHB, Evmenenko G, Nguyen ST, Ruoff RS (2007). Preparation and characterization of graphene oxide paper. Nature.

[40] Tian T, Shi X, Cheng L, Luo Y, Dong Z, Gong H, Xu L, Zhong Z, Peng R, Liu Z (2014). Graphene-based nanocomposite as an effective, multifunctional, and recyclable antibacterial agent. ACS Appl Mater Interfaces.

[41] Tao B, Lin C, Deng Y, Zhang Y, Cai K (2019). Copper-nanoparticle-embedded hydrogel for killing bacteria and promoting wound healing with photothermal therapy. J Mater Chem B.

[42] Xu ZQ, Wang X, Liu X, Cui Z, Yang X, Yeung KWK, Chung JC, Chu PK, Wu S (2017). Tannic acid/Fe^3+^/Ag nanofilm exhibiting superior photodynamic and physical antibacterial activity. ACS Appl Mater Interfaces.

[43] Ping L, Sun S, Dong A, Hao Y, Shi S, Sun Z, Ge G, Chen Y (2015). Developing of a novel antibacterial agent by functionalization of graphene oxide with guanidine polymer with enhanced antibacterial activity. Appl Surf Sci.

[44] Kellici S, Acord J, Vaughn A, Power NP, Morgan DJ, Heil T, Facq SP, Lampronti GI (2016). Calixarene assisted rapid synthesis of silver-graphene nanocomposites with enhanced antibacterial activity. ACS Appl Mater Interfaces.

[45] Wu Y, Long Y, Li QL, Han S, Ma J, Yang YW, Gao H (2015). Layer-by-layer (LBL) self-assembled biohybrid nanomaterials for efficient antibacterial applications. ACS Appl Mater Interfaces.

