# Supplementary material for: Construction of biocompatible bovine serum albumin nanoparticles composed of nano graphene oxide and AIEgen for dual-mode phototherapy bacteriostatic and bacterial tracking
Source: J Nanobiotechnology. 2019 Oct 10;17:104. doi: 10.1186/s12951-019-0523-x (PMC6785860; doi:10.1186/s12951-019-0523-x)
Supplement: Supplementary file 1 — Additional file 1. Additional experimental section and additional figures. [file 12951_2019_523_MOESM1_ESM.docx]

Additional fle

**Construction of Biocompatible Bovine Serum Albumin Nanoparticles Composed of Nano Graphene Oxide and AIEgen for Dual-mode Phototherapy Bacteriostatic and Bacterial Tracking**

*Yongxin Zhang, Hao Fu, De-E Liu, Jinxia An,* Hui Gao**

School of Chemistry and Chemical Engineering, Tianjin Key Laboratory of Organic Solar Cells and Photochemical Conversion, Tianjin University of Technology, No. 391, West Binshui Road, Tianjin 300384 (P. R. China).

E-mail: hgao@tjut.edu.cn (H.G.); anjx2016@email.tjut.edu.cn (J.A.)

Table of Contents

**1. Experimental Section3**

**1.1** **Materials and Instruments3**

**1.2 Synthesis of Nano Graphene Oxide (NGO)4**

**1.3 Synthesis of (2Z,2'Z)-3,3'-(2,5-bis(phenyl(4-((tetrahydro-2H-pyran-2-yl)oxy)phenyl) amino)-1,4-phenylene)bis(2-(3,5-bis(trifluoromethyl)phenyl)acrylonitrile) (3) (AIEgen)4**

**1.4 DLS Measurements of NGO and NGO-BSA-AIE NPs 5**

**1.5 Bacterial Culture5**

**1.6 Detection of ROS in Bacteria6**

**1.7 Scanning Electron Microscopic (SEM)6**

**1.8 Confocal Microscopy Observations7**

**1.9 Cell Culture and Imaging 7**

**1.10 Statistics Analysis8**

**2. Figures 8**

**3. References 12**

**1. Experimental Section**

**1.1 Materials and Instruments**

Graphite (flakes, 99% carbon basis, 325 mesh particle size (≥99%), natural) was obtained from Sigma-Aldrich (Shanghai, China). The concentrated H_2_SO_4_ (98%), potassium permanganate (KMnO_4_), potassium tetraphosphate (K_3_PO_4_), chromium trioxide (CrO_3_), HCl (38%), NaOH, acetic acid, acetic anhydride, dichloromethane, toluene, tetrahydrofuran (THF), methanol and ethanol were purchased from Tianjin Chemical Reagent Co. (Tianjin, China), and H_2_O_2_ (30%) was bought from Aladdin (Shanghai, China). 4’,6-Diamidino-2-phenylindole (DAPI), 2',7'-dichlorofluorescein diacetate (DCF-DA), 3,5-bis(trifluoromethyl)phenylacetonitrile, bis(dibenzylideneacetone)palladium(0) [Pd_2_(dba)_3_], 2-dicyclohexylphosphino-2',6'-diisopropoxybiphenyl (Ruphos) were purchased from Energy Chemical Co., Ltd. (Shanghai, China). Sodium nitrate (NaNO_3_), bovine serum albumin (BSA), 1,4-dibromo-2,5-dimethylbenzene, ethyl bromoacetate, N-pheny-4-(tetrahydro-2H-pyran-2-yi)oxy)aniline, 4-hydroxydiphenylamine, and pyridinium p-toluenesulfonate were purchased from Heowns (Tianjin, China). 3-(4,5-Dimethyl thiazol-2-yl)-2,5-diphenyltetrazolium bromide (MTT) was used in the cell cytotoxicity assay (Dojindo, Japan).

Fourier transform infrared (FT-IR) spectra were obtained from a Bio-Rad 6000 spectrophotometer (Thermo Electron, USA) using potassium bromide pellets. The crystalline structure of samples was determined by X-ray diffraction (XRD) on a diffractometer (Dmax 2500PC Rigaku, Japan) with Cu Kα (λ = 1.5406 Å), radiation at a voltage of 40 kV and a current of 100 mA. The samples were scanned with a scanning speed of 3°/min from 5°-80°. ^1^H-NMR spectra of AIEgen derivatives was recorded on a 400 MHz Bruker Avance-400 spectrometer (400 MHz, Bruker, Freemont, CA). The chemical shifts were referred to the solvent peaks, δ = 2.50 ppm for DMSO. UV-Vis spectra were recorded on a Nanophotometer NP80 Touch spectrophotometer. Fluorescence spectra were carried out with a Hitachi F4500 spectrofluorophotometer and Maya2000Pro optical fiber spectrophotometer. A field emission scanning electron microscope (FESEM, JEOL JSM-6700F) was applied to observe the morphology of nanoparticles and bacteria. Bacterial imaging was observed by confocal laser scanning microscopy (Nikon A1 CLMS). Dynamic light scattering (DLS) measurements were performed using a Malvern Nano ZS90 instrument at 25 ^o^C.

**1.2 Synthesis of Nano Graphene Oxide (NGO)**

GO was prepared using the improved Hummer's method.^[1]^ Graphite was slowly added to 50 mL of concentrated H_2_SO_4_ in ice bath conditions and then stirred for 6 hours at 25 ^o^C. Next, in ice bath conditions, NaNO_3_ (1.0 g, 11.7 mmol) was put into the mixture and continued to stir for 30 minutes. Under stirring, KMnO_4_ (3.0 g, 18.9 mmol) was added slowly to the mixture (0.5 g x 6) in an ice bath. Subsequently, the mixture was kept at 25 ^o^C and stirred constantly for 3 days. After that, 50 mL of HCl (5%) was added into the solution slowly and the mixture was heated to 98 °C and stirred for another 30 minutes. After cooling, H_2_O_2_ was dropped into the mixture in order to remove metal ions, and the color of mixture turned yellow. Finally, GO was harvested by centrifugation at 5000 rpm for 5 minutes and washed with deionized water three times, and then drying in a freeze dryer. The obtained GO was dispersed in deionized water and sonicated for 4 hours. Then, the solution was filtered through a microporous membrane (0.22 μm). The GO nanoparticle filtrate was collected and stored for further use.

**1.3** **Synthesis of (2Z,2'Z)-3,3'-(2,5-bis(phenyl(4-((tetrahydro-2H-pyran-2-yl)oxy)phenyl) amino)-1,4-phenylene)bis(2-(3,5-bis(trifluoromethyl)phenyl)acrylonitrile) (3)** **(AIEgen)**

Compound 1 and 2 were synthesized according to the previous literature.^2-4^ In brief, compound 1 (0.227 g, 0.298 mmol), compound 2 (0.802 g, 2.747 mmol), K_3_PO_4_ (0.632 g, 2.974 mmol), Pd_2_(dba)_3_ (0.027 g, 0.028 mmol), Ruphos (0.038 g, 0.081 mmol), and toluene (10.0 mL) were added to a two-necked flask under N_2_ atmosphere with magnetic stirring at 110 ^o^C for 24 hours. After the reaction mixture was cooled to room temperature 60.0 mL water and 100 mL chloroform were added. An organic layer was separated and washed with brine, dried over anhydrous MgSO_4_ and evaporated to dryness under reduced pressure. The crude product was purified by silica gel chromatography (petroleum:ethyl acetate = 5:1), and then recrystallized from CH_2_Cl_2_/ethanol to give compound 3 (AIEgen). Yield: 68.4%. ^1^H NMR (400 MHz, DSMO-d6, TMS, ppm): δ 8.12–8.14 (d, J = 8.4 Hz, 4H), 7.88 (s, 4H), 7.59 (s, 2H), 7.22–7.26 (t, J = 8.0 Hz, 4H), 7.05–7.07 (d, J = 8.8 Hz, 4H), 6.99–7.01 (d, J = 8.0 Hz, 4H), 6.90–6.93 (m, 6H), 5.22–5.23 (t, J = 6.4 Hz, 2H), 3.59–3.64 (m, 2H), 3.31–3.43 (m, 2H), 1.47–1.88 (m, 12H).


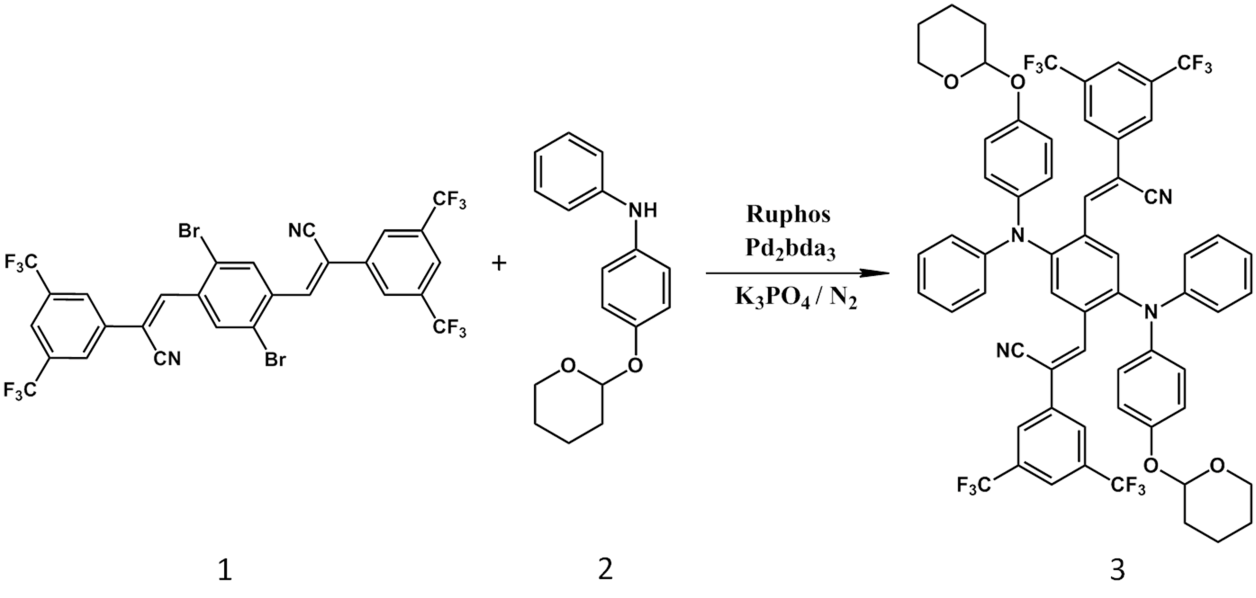


**Scheme S1.** Synthetic route of the compound 3 (AIEgen).

**1.4 DLS Measurements of NGO and NGO-BSA-AIE NPs**

The size of NGO and NGO-BSA-AIE NPs were determined by Zetasizer Nano ZS90 instrument (Malvern Instruments, Southborough, MA) at 25 ^o^C.

**1.5** **Bacterial Culture**

(AMO)-resistant *Escherichia coli* (*E. coli*) and *Staphylococcus aureus* (*S. aureus*) were employed as the representative bacteria for testing the antibacterial activities of the proposed NGO-BSA-AIE NPs. In brief, the bacteria were cultured into liquid Luria-Bertani (LB) medium (5 mL) on a shaking incubator (37 °C, 170 rpm) overnight. To create resistant variants, the cultures were passaged 30 times in the presence of rising concentrations of AMO based on the growth conditions of the variants. Then, the concentration of AMO^r^ *E. coli* and AMO^r^ *S. aureus* were quantiﬁed by measuring optical density (OD) at λ = 600 nm (OD_600_ of 2.0 was determined to have a concentration of 10^9^ colony forming units per milliliter (CFU mL^-1^).^5^

**1.6 Detection of ROS in** **Bacteria**

The DCF-DA was used as an indicator to detect the production of ROS in bacteria under the irradiation of daylight by confocal laser microscopy system (CLMS). AMO^r^ *E. coli* and AMO^r^ *S. aureus* were incubated at 37 ^o^C overnight to a concentration of 10^9^ CFU mL^-1^ and washed with PBS three times, followed by being transferred to a 35 mm glass-bottom after proper dilution. Following incubation with NGO-BSA-AIE NPs (NGO: 0.5 mg mL^-1^, BSA: 2 mg mL^-1^, AIE: 10 μg mL^-1^, 100 μL) for 6 hours in the dark, the bacteria were rinsed with PBS for three times and stained with 10 μM of DCF-DA. After 20 minutes incubation, the bacteria were washed three times with PBS and then irradiated with daylight for different time intervals. After irradiation, the bacteria were observed by CLMS. Pertaining to NGO-BSA-AIE NPs detection, the excitation wavelength was 488 nm while the fluorescence emission range was collected at 598-662 nm; for DCF-DA detection, the excitation wavelength was 488 nm while fluorescence emission range was collected at 500-530 nm.^4^

**1.7 Scanning Electron Microscopic (SEM)**

In order to gain insight into the results of the photothermal and photodynamic effects of NGO-BSA-AIE NPs on bacterial antibacterial activity, SEM was used in this study. Here, the AMO^r^ *E. coli* and AMO^r^ *S. aureus* were incubated at 37 ^o^C to a concentration of 10^9^ CFU mL^-1^. The bacteria were then harvested by centrifugation and resuspended in PBS (pH = 7.4). Then bacterial suspensions alone, bacterial suspensions that exposed to daylight for one hour and NIR laser for 5 minutes, bacterial suspensions treated with NGO-BSA-AIE NPs (in a dark environment, or irradiated with daylight for one hour, or irradiated with 795 nm NIR laser for 5 minutes, or irradiated with daylight for one hour followed by being exposed to NIR laser for 5 minutes, respectively) were shaken at 170 rpm, 37 ^o^C for 16 hours, followed by being centrifuged at 8000 rpm for 5 minutes and washed three times with PBS. The samples were then further washed three times with PBS and dehydrated by addition to a graded series of ethanol solutions (30%, 50%, 70%, 85%, 95% and 100%, 30 min), followed by the addition of isoamyl acetate. Finally, the samples were dropped onto the silicon wafer and air dried for SEM analysis.^5,6^

**1.8 Confocal Microscopy Observations**

The bacteria (AMO^r^ *E. coli* and AMO^r^ *S. aureus*) incubated overnight at 37 °C that reached the concentration of 10^9^ CFU mL^-1^ were harvested by centrifugation (8000 rpm, 3 min) and washed with PBS (pH = 7.4) for three times. The supernatant was removed and the remaining bacteria were resuspended in 1 mL of PBS. Then the bacteria alone or treated with the NGO-BSA-AIE NPs that after being irradiated by daylight and NIR laser were shaken at 170 rpm at 37 °C for 16 hours and stained with 100 μL acridine orange (AO, 1 mg mL^-1^) and ethidium bromide (EB, 1 mg mL^-1^) fluorescent dye for 30 minutes. Then, the bacterial cells were washed with PBS three times and imaged by CLSM. The live and dead bacterial cells were excited at 488 nm, 514 nm and the emission was collected at 500-530 nm, 552-617 nm, respectively.

**1.9 Cell Culture and Imaging**

L929 cell lines were incubated in RPIM 1640 media (Thermo Fisher Scientific from Shanghai, China) supplemented with 10% fetal bovine serum (FBS) and 1% antibiotics in humidified environment containing 5% CO_2_ at 37 °C. CLSM was performed to explore the internalization of NGO-BSA-AIE NPs. L929 cells were seeded at a density of 10^5^ cells onto a 35 mm glass dish and incubated for 24 hours in 400 μL of RPIM 1640 medium supplemented with 10% FBS. The adherent cells were washed twice with PBS buffer before the experiment. NGO-BSA-AIE NPs (NGO: 0.5 mg mL^-1^, BSA: 2 mg mL^-1^, AIE: 10 μg mL^-1^, 100 μL) were then added to the plates. After 6 hours of incubation, the cells were washed three times with PBS buffer, and the cells were fixed with 1 ml of 75% ethanol for 20 minutes, and then the cells were washed three times with PBS buffer to remove the alcohol. Next, 200 μL of DAPI was added, stained for 30 minutes, and then the stained cells were washed three times with PBS buffer, and finally the cells were retained in 1 mL of PBS for bioimaging. For DAPI: λex = 402 nm, λem = 461 nm.

**1.10 Statistics Analysis.** Significant differences in antibacterial activity assay were evaluated using Student’s *t*-test.

**2. Figures**

XRD spectral analysis of graphite and GO are shown in Figure S1a. The XRD characterization results showed that the diffraction peak of graphite was 2θ = 26.4^o^, indicating that the corresponding interlayer distance was 0.34 nm. For GO, a narrow and high diffraction peak with a 2θ = 10.5^o^ appeared after the oxidation reaction with a corresponding layer spacing of 0.86 nm because oxygen-containing functional groups were formed on the surface of the GO, demonstrating that GO was successfully synthesized. FT-IR spectra characterization results indicated that the spectrum of GO demonstrated C−O−C stretching vibrations around 1276 cm^-1^, the C = C bond at 1625 cm^-1^, the C = O stretching vibration at 1725 cm^-1^, the asymmetric stretching of the C-H bond at 2927 cm^-1^, and the O-H stretching vibration at 3422 cm^-1^, respectively (Figure S1b). In addition, SEM imaging of the GO sheets surface morphology was shown on Figure S1c. The GO was composed of agglomerated stacked nanosheets and the monolayer thickness of GO was about 25 nm.


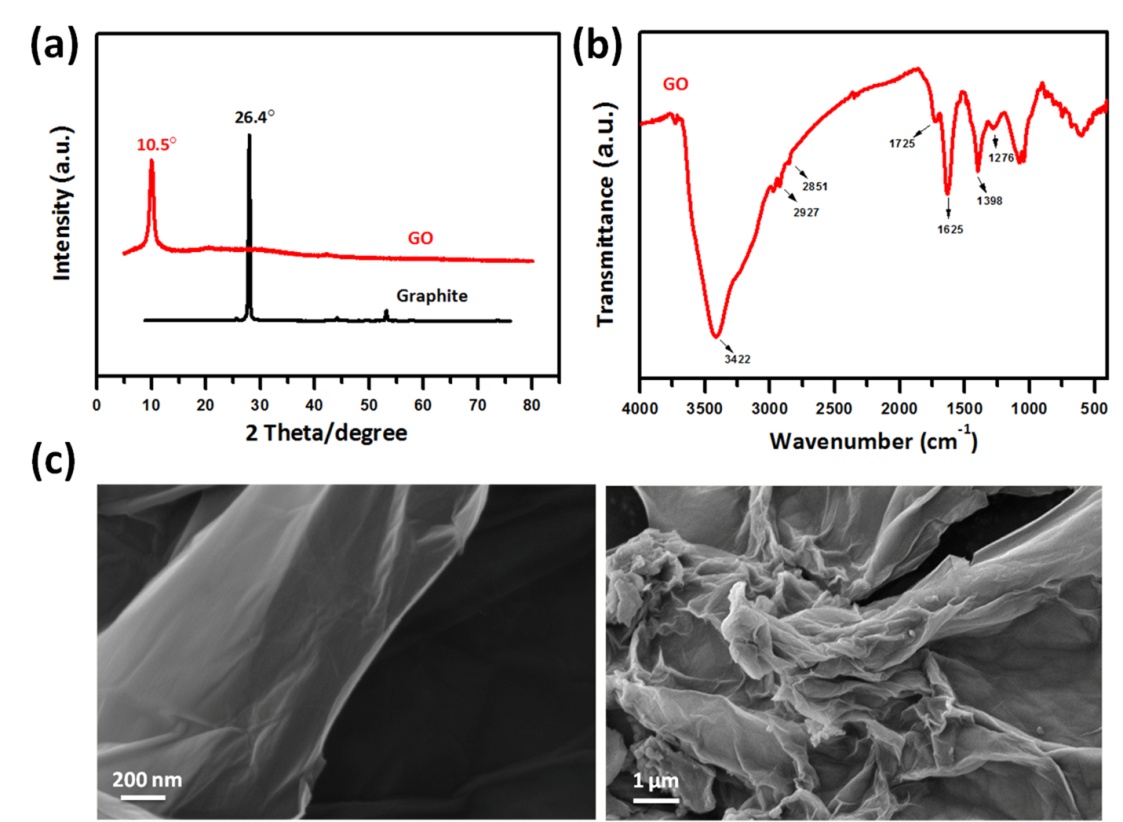


**Figure S1.** (a) XRD patterns of graphite and GO, (b) FT-IR spectrum characterization of GO

and (c) SEM images of GO sheets.


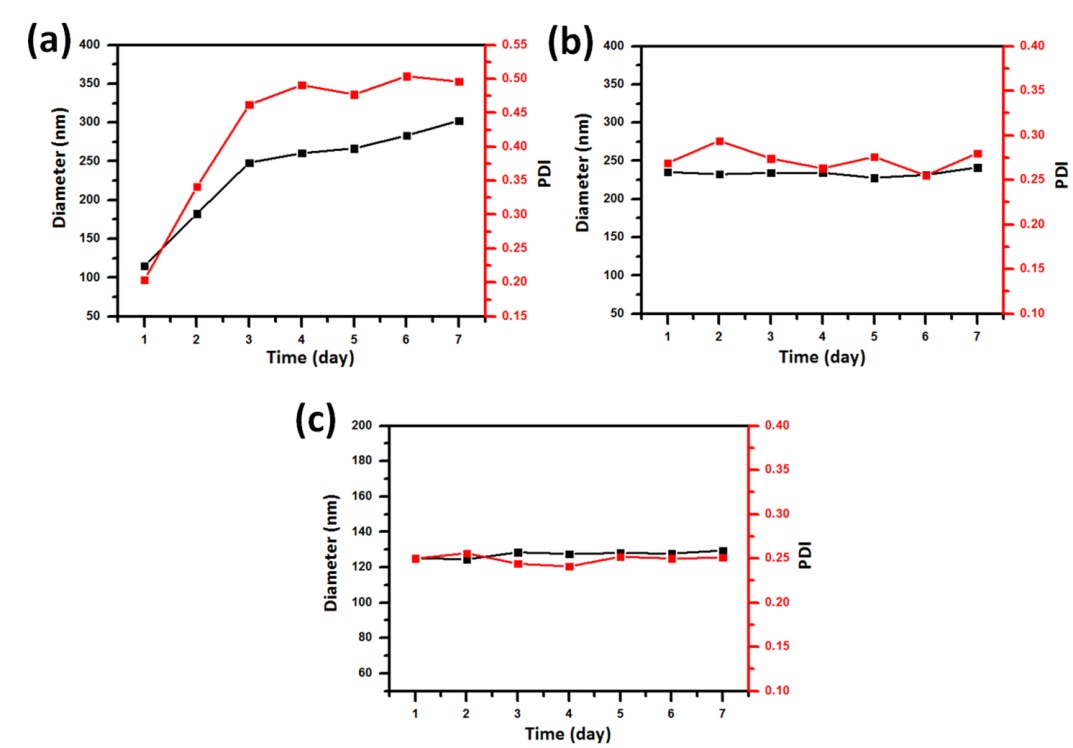


**Figure S2.** Stability evaluation of (a) NGO and (b) NGO-BSA-AIE NPs within 7 days in PBS

and (c) NGO-BSA-AIE NPs within 7 days in water by DLS size monitoring.

**
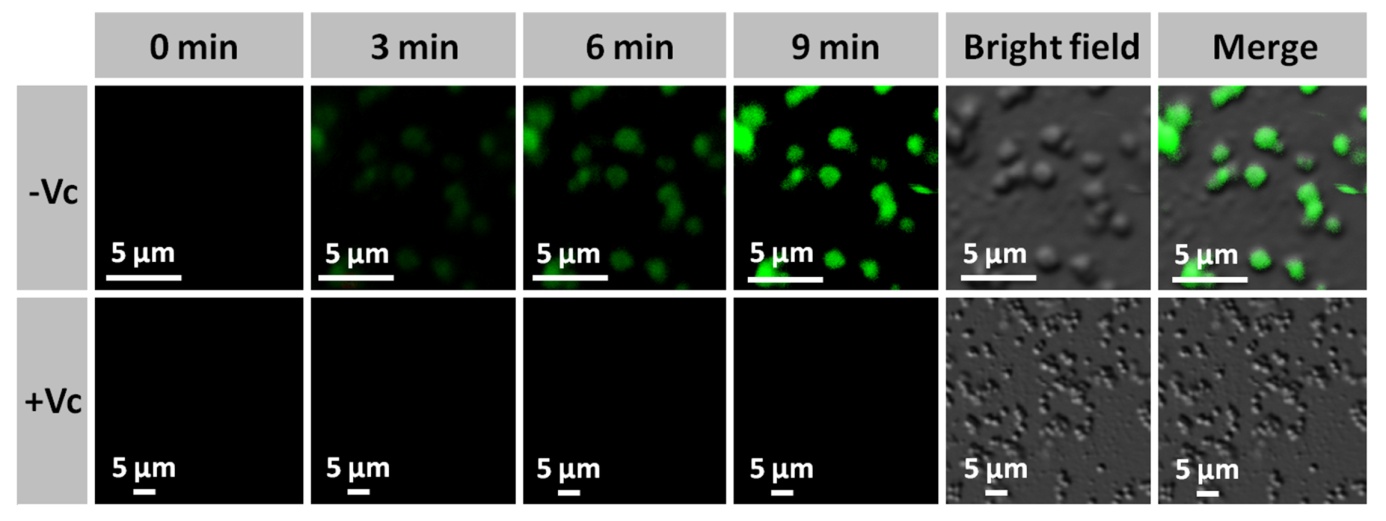
**

**Figure S3.** CLSM imaging of AMO^r^ *S. aureus* after incubation with NGO-BSA-AIE NPs and

DCF-DA under daylight irradiation for different time in the presence and absence of vitamin

C. Scale bars: 5 μm.

**
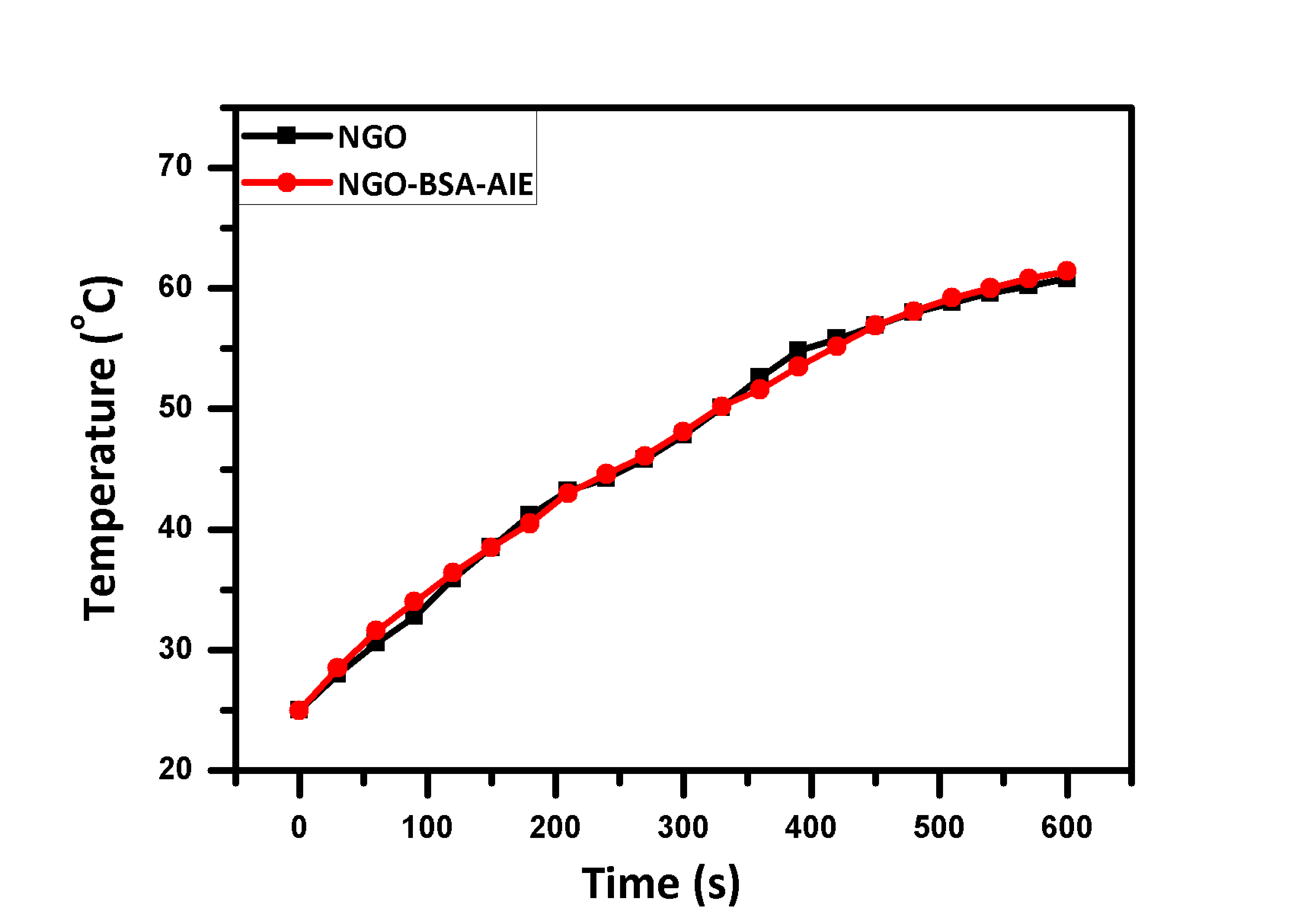
**

**Figure S4.** Photothermal curves of NGO and NGO-BSA-AIE NPs for 10 minutes under 795

nm NIR laser irradiation. NGO: 0.5 mg mL^-1^.

**
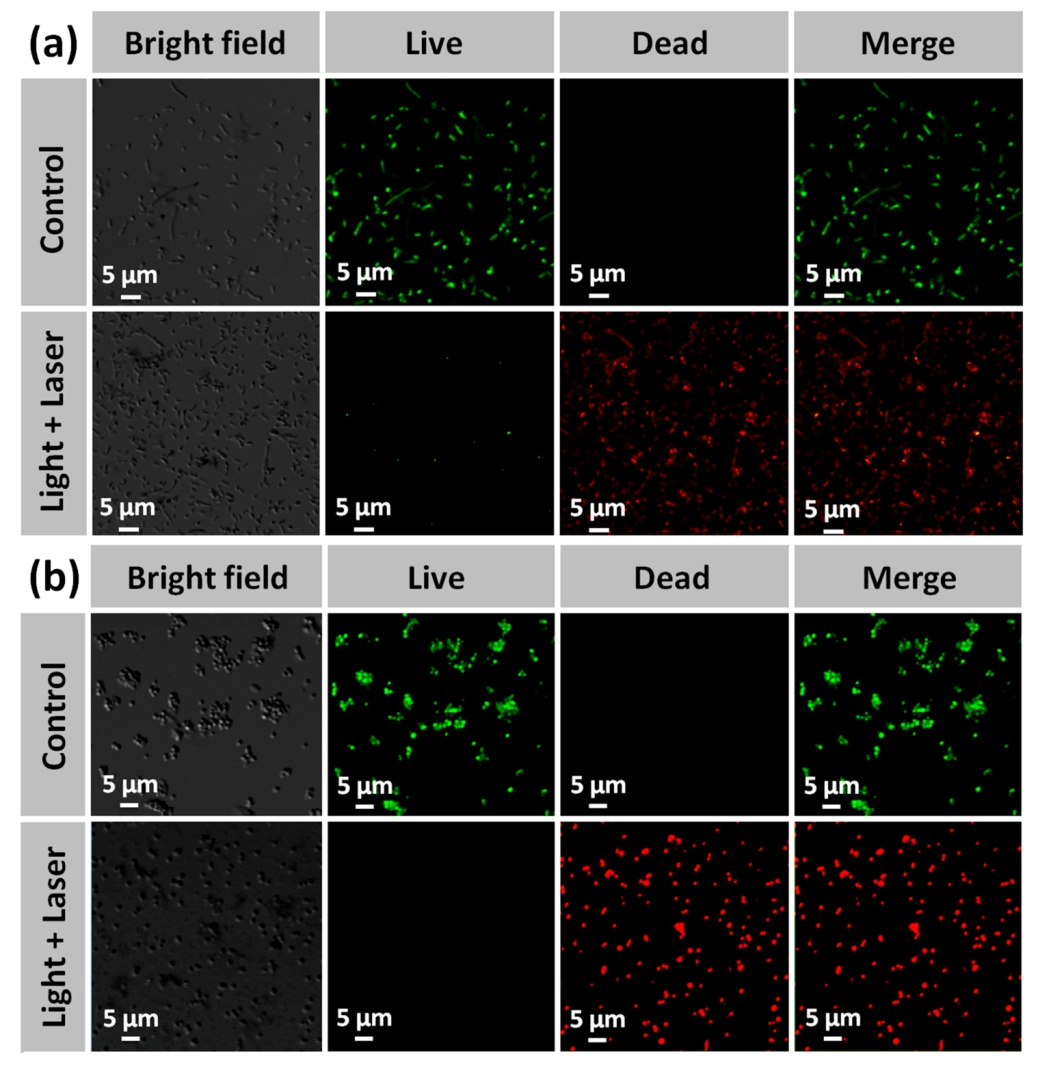
**

**Figure S5.** CLSM images of (a) AMO^r^ *E. coli* and (b) AMO^r^ *S. aureus* treated with PBS and

NGO-BSA-AIE NPs that exposed to daylight followed by 795 nm NIR laser. Scale bars: 5


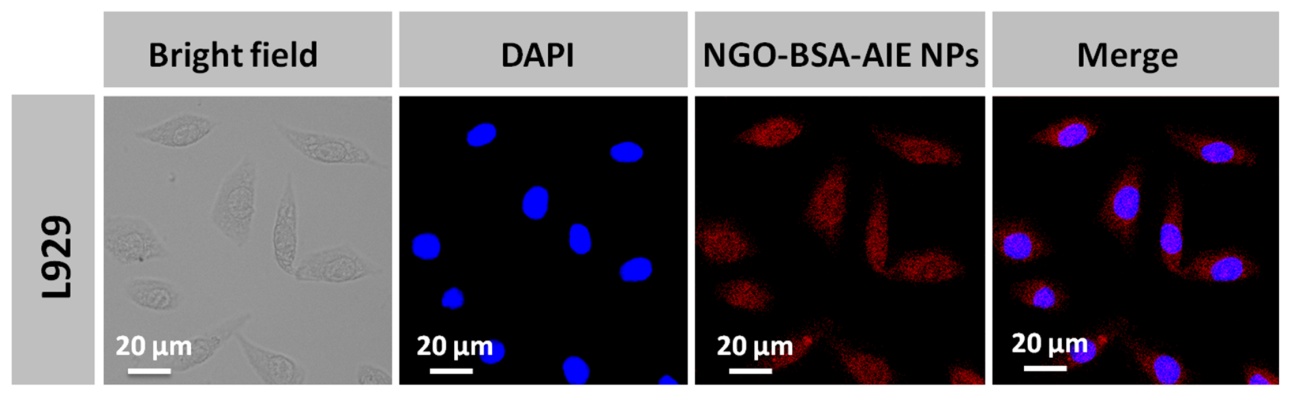
µm.

**Figure S6.** Intracellular distribution observed by CLSM of NGO-BSA-AIE NPs in L929 cells.

Scale bars: 20 μm.

**3. References**

1. Dikin DA, Stankovich S, Zimney EJ, Piner RD, Dommett GH B, Evmenenko G, Nguyen ST, Ruoff RS. Preparation and characterization of graphene oxide paper. Nature. 2007;448:457-60.

2. Lu H, Zheng Y, Zhao X, Wang L, Ma S, Han X, Xu B, Tian W, Gao H. Highly efficient far red/near-infrared solid fluorophores: aggregation-induced emission, intramolecular charge transfer, twisted molecular conformation, and bioimaging applications. Angew Chem Int Ed Engl. 2016;55:155-9.

3. Guan Y, Lu H, Li W, Zheng Y, Jiang Z, Zou J, Gao H. Near-infrared triggered upconversion polymeric nanoparticles based on aggregation-induced emission and mitochondria targeting for photodynamic cancer therapy. ACS Appl Mater Interfaces. 2017;9:26731-9.

4. Huang Y, Chen Q, Lu H, An J, Zhu H, Yan X, Li W, Gao H. Near-infrared AIEgen-functionalized and diselenide-linked oligo-ethylenimine with self-sufficing ROS to exert spatiotemporal responsibility for promoted gene delivery. J Mater Chem B. 2018;6:6660-6.

5. Chen S, Chen Q, Li Q, An J, Sun P, Ma J, Gao H. Biodegradable synthetic antimicrobial with aggregation-induced emissive luminogens for temporal antibacterial activity and facile bacteria detection. Chem Mater. 2018;30:1782-90.

6. Li Q, Wu Y, Lu H, Wu X, Chen S, Song N, Yang YW, Gao H. Construction of supramolecular nanoassembly for responsive bacterial elimination and effective bacterial detection. ACS Appl Mater Interfaces. 2017;9:10180-9.
